# Prognostic Value of Cancer Stem Cell Marker CD133 Expression in Gastric Cancer: A Systematic Review

**DOI:** 10.1371/journal.pone.0059154

**Published:** 2013-03-22

**Authors:** Lei Wen, Xin-Zu Chen, Kun Yang, Zhi-Xin Chen, Bo Zhang, Jia-Ping Chen, Zong-Guang Zhou, Xian-Ming Mo, Jian-Kun Hu

**Affiliations:** Department of Gastrointestinal Surgery, West China Hospital, Sichuan University, Chengdu, Sichuan Province, China; University of Kansas Medical Center, United States of America

## Abstract

**Objective:**

To investigate the correlation between CD133-positive gastric cancer and clinicopathological features and its impact on survival.

**Methods:**

A search in the Medline and Chinese CNKI (up to 1 Dec 2011) was performed using the following keywords gastric cancer, CD133, AC133, prominin-1 etc. Electronic searches were supplemented by hand searching reference lists, abstracts and proceedings from meetings. Outcomes included overall survival and various clinicopathological features.

**Results:**

A total of 773 gastric cancer patients from 7 studies were included. The median rate of CD133 expression by immunohistochemistry (IHC) was 44.8% (15.2%–57.4%) from 5 studies, and that by reverse transcription polymerase chain reaction (RT-PCR) was 91.3% (66.7%–100%) from 4 studies. The accumulative 5-year overall survival rates of CD133-positive and CD133-negative patients were 21.4% and 55.7%, respectively. Meta-analysis showed that CD133-positive patients had a significant worse 5-year overall survival compared to the negative ones (OR = 0.20, 95% CI 0.14–0.29, P<0.00001). With respect to clinicopathological features, CD133 overexpression by IHC method was closely correlated with tumor size, N stage, lymphatic/vascular infiltration, as well as TNM stage.

**Conclusion:**

CD133-positive gastric cancer patients had worse prognosis, and was associated with common clinicopathological poor prognostic factors.

## Introduction

Gastric cancer (GC) is the fourth most common cancer worldwide [1]. Following only lung cancer, GC is also the second leading cause of cancer-related death in Asia. Although underwent radical resection and postoperative adjuvant therapy, most of GC patients will die of recurrence and metastasis, with 5-year overall survival no more than 50% for resectable patients in China [2].

The advancement in survival of GC patients in past few decades was relatively small, due to a lack of deep understanding the molecular mechanism of cancer. Recently, a rare subpopulation cancer cells, termed cancer stem cells (CSC) have been thought to be responsible for the initial, progression, metastasis and ultimately recurrence of cancer, for they have the exclusive properties of self-renew and could giving rise to all the heterogeneous lineages of cancer cells that eventually constitute tumor bulk [3]. CD133 is a trans-membrane glycoprotein, its expression in cell surface down-regulates quickly as cell differentiated [4]. CD133 has been used widely as a marker to identify CSC in colon, lung, brain, pancreatic cancer and so on [5]–[8]. Its prognostic value for cancer patients has also been found in many cancers [9]–[12].

With respect to gastric cancer, the correlation between CD133 and clinicopothological features of GC and its prognostic value is relatively unclear. Thus a systematic review of published literatures was conducted to clarify the relationship between CSC marker CD133 and GC based on current evidences.

## Methods

### Literature Search Strategy

A comprehensive literature search of electronic databases PubMed and Chinese CNKI was performed up to December 1, 2011. Search strings of PubMed was (((“cd133” [Title/Abstract]) OR “ac133” [Title/Abstract]) OR “prominin 1” [Title/Abstract]) AND (((“stomach neoplasms” [MeSH Terms]) AND “carcinoma” [MeSH Terms]) OR “gastric cancer” [Title/Abstract]). The reference lists of relative articles were also screened to further identify potential studies.

### Selection Criteria

Diagnosis of gastric cancer was proven by histopathological methods. Studies of CD133 expression based on primary gastric cancer tissue (via either surgical or biopsy), rather than serum or any other kinds of specimen were included. Expression of CD133 was detected by any method. All studies on the correlation of CD133 overexpression with clinicopathological markers and the association of CD133 overexpression on disease-free and overall survival of gastric cancer were included. There was no limitation on language as well as the minimum patients of every single study. When there were multiple articles by the same group based on similar patients and using same detection methods, only the largest or the most recently article was included.

### Data Extraction

Data tables were made to extract all relevant data from texts, tables and figures of each included studies, including author, year, country, patient number, detection method, clinicopathological features, positive rates of CD133 overexpression, as well as the expression-related survival. In case the prognosis was only plotted as Kaplan-Meier curve in some articles, the software GetData Graph Digitizer 2.24 (http://getdata-graph-digitizer.com/) was applied to digitize and extract the data.

### Statistical Analysis

Meta-analysis was supplemented when applicable; otherwise, outcomes were presented in a narrative way. Software RevMan 5.0 (the Cochrane Collaboration, Copenhagen) was employed for data analysis. Comparisons of dichotomous measures were performed by pooled estimates of odds ratios (OR), as well as their 95% confidence intervals (CI). P value<0.05 was considered as statistical significance. Fixed or Random model was used depending on heterogeneity analysis. Statistical heterogeneity was tested using a Chi-square test with significance being set at p<0.10, the total variation among studies was estimated by I-square [13].

## Results

### Literatures Information

**Figure 1:**
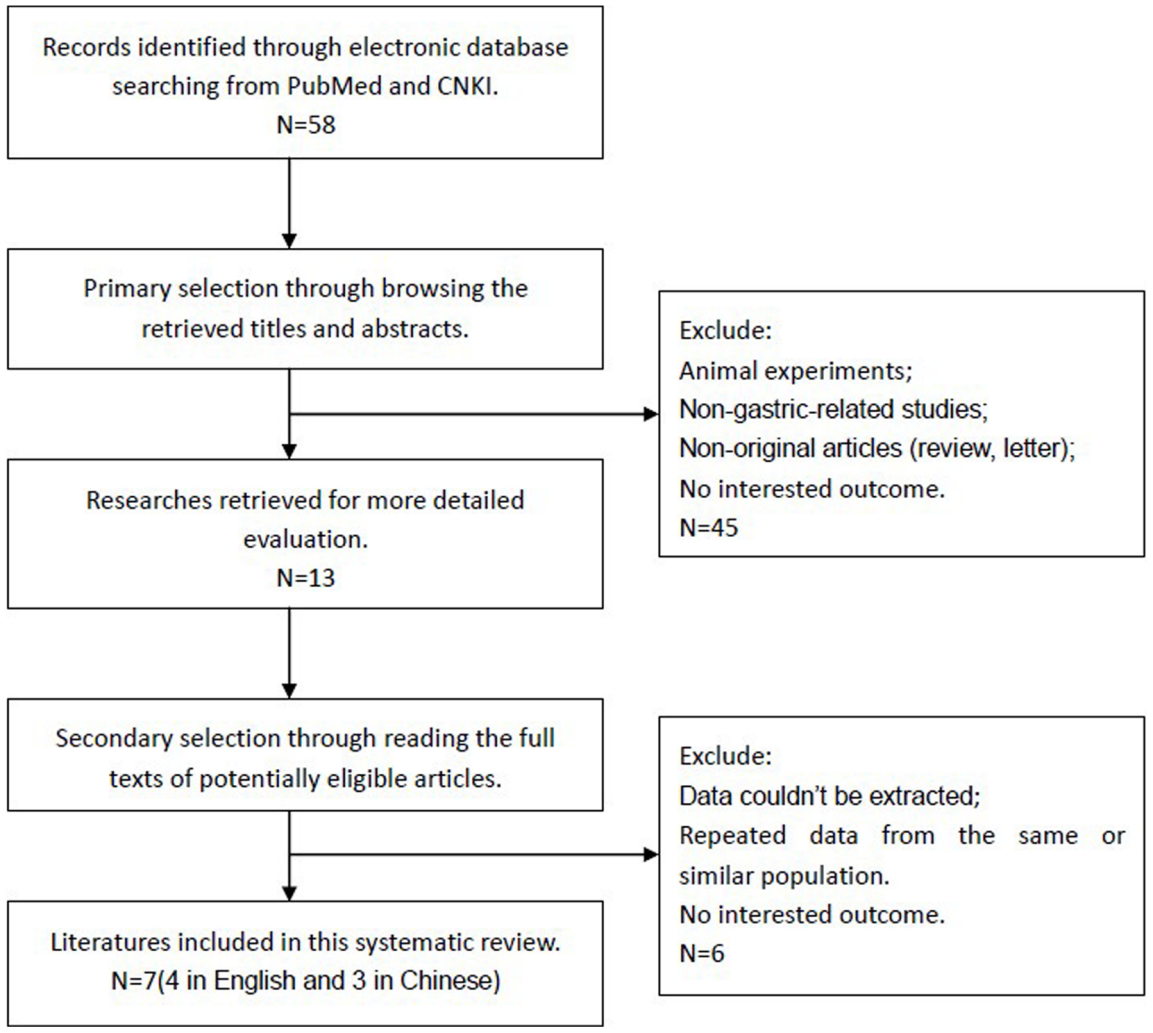
Flow chart for selection of studies.

Fifty-eight articles were identified initially using the search strategy above. Forty-five of those were excluded due to non-human experiments, non-gastric-related studies, non-original articles (review, letter), through reading title and abstract. After excluded those data couldn’t be extracted and repeated ones by reading full text [14]–[19], eventually, there were 7 studies (4 in English and 3 in Chinese) included in the present Meta-analysis [20]–[26] (Figure 1).

### Study Characteristics

The 7 included studies were all based on Asian population, including 1 from Japan, 1 from Singapore and the rest 5 from China. A total of 773 patients with a median of 110 (ranged from 31 to 336) were included, most of which were male patients (74.8% from 6 studies). The median age ranged from 58 to 65 years old. In regarding to TNM stage, a median of 43.2% (22.6%–72.2%) patients were stage I or II, while the other 56.8% (27.8%–77.4%) were stage III or IV. Differentiated grading of tumor was reported in 5 studies and among those, roughly two thirds were poorly differentiated. Around 67.8% (58.8%–78.9%) of reported patients were identified as metastatic lymph node status.

### CD133 Expression Status Stratification

All detected specimen were derived from gastric cancer tissues by means of surgical resection. Two different methods were employed for CD133 overexpression status screening. In detailed, immunohistochemistry (IHC) on membrane protein level is involved in 3 included studies, reverse transcription polymerase chain reaction (RT-PCR) on mRNA level in 2 studies and the both in the rest two studies. The median rate of CD133 overexpression by IHC from 5 studies was 44.8% (15.2%–57.4%). CD133 mRNA overexpression rates in gastric cancer tissue were much higher, with a median rate of 91.3% (66.7%–100%), which was of significant differences compared to normal gastric tissue in all 4 studies according to RT-PCR results (Table 1).

**Table 1:** General characteristics of included studies.

Studies	Year	Cases	Method	CD133 expression rate (%)	5-year OS (%)		P value
					CD133 (+)	CD133 (−)	
Wang T [26]	2011	103	IHC	44	20.8	43.4	<0.01
Zhao P [23]	2010	336	IHC	57.4	22.8	57.3	<0.01
Ishigumin S [24]	2010	97	IHC	28	29	78	<0.01
Yu JW [25]	2010	99	IHC	29.3	0	40.4	<0.01
		31	RT-PCR	100	–	–	–
Xue YM [22]	2011	33	IHC	15.2	–	–	–
		33	RT-PCR	100	–	–	–
Tang BF [20]	2008	38	RT-PCR	100	–	–	–
Yan JL [21]	2011	36	RT-PCR	66.7	–	–	–
***Summary***	**–**	**742**	**IHC**	**44.8%**	**21.4**	**55.7**	**–**
			**RT-PCR**	**91.3%**			

**Abbreviations:** OS, over survival; GC, gastric cancer; IHC, immunohistochemistry; RT-PCR, reverse transcription polymerase chain reaction.

### CD133 Overexpression and 5-year Overall Survival

**Figure 2:**
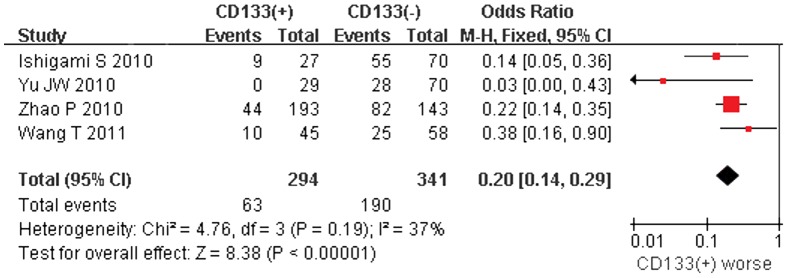
Meta-analysis of 5-year overall survival between CD133(+) and CD133(−) gastric cancer patients.

5-year overall survival rate was extracted from 4 studies, all of which relied on IHC solution. The accumulative 5-year overall survival rates of CD133-positive and CD133-negative GC patients were 21.4% (63/294) and 55.7% (190/341), respectively. (Table 1) Meta-analysis indicated that patients with CD133-positive suffered with a significant poor prognosis in comparing to CD133-negative ones (OR = 0.20, 95% CI: 0.14–0.29, P<0.00001, Fixed Model). In fact, 3 out of 4 studies have also concluded CD133 overexpression as a poor prognostic factor in gastric cancer patients (Figure 2).

### CD133 Overexpression and Clinicopothological Features

All seven studies presented data on clinicopothological features. However, two different screening solutions (IHC and RT-PCR) are applied and variance also appeared in criterions, i.e., for tumor size, Zhao et al. taken 4 cm as borderline while Yu et al. used 5 cm, therefore, systematic review was conducted in a narrative way instead of meta-analysis.

In gastric cancer patients, there was no clear correlation between CD133 overexpression and ages (5 out of 5 studies), sex (4 out of 4 studies), tumor depth (5 out of 7 studies) and tumor grades (5 out of 7 studies). However, CD133 overexpression was likely to associate with tumor size (4 out of 5 studies), lymph node metastasis (5 out of 7 studies) as well as lymphatic vessel/vascular infiltration (3 out of 3 studies). With regard to the relationship between TNM stages and CD133 overexpression, unexpected, an opposite trend has been found in two different screening solutions. Three out of four studies by IHC revealed a positive correlation between CD133 and tumor stages whereas 4 studies by RT-PCR suggested none (Table 2).

**Table 2:** Narrative review of clinicopathological features with CD133 overexpression.

Items	Significant correlation (p<0.05)	Non-significant correlation (p≥0.05)
Age	–	IHC: [23], [25], [26] RT-PCR: [21], [22], [25]
Sex	–	IHC: [25], [26] RT-PCR: [21], [22], [25]
Tumor Size	IHC: [23], [25] RT-PCR: [20], [22]	RT-PCR: [21]
Depth	IHC: [23], [24]	IHC: [25], [26] RT-PCR: [20], [21], [22], [25]
TNM stage	IHC: [23], [24], [25]	IHC: [26] RT-PCR: [20], [21], [22], [25]
Tumor grade	IHC: [23] RT-PCR: [21]	IHC: [24], [25], [26] RT-PCR: [20], [22], [25]
Lymph node metastasis	IHC: [23], [24], [25] RT-PCR: [20], [22], [25]	IHC: [26] RT-PCR: [21]
TNM stage	IHC: [23], [24], [25]	IHC: [26] RT-PCR: [20], [21], [22], [25]
Lymphatic vessel/vascular infiltration	IHC: [24], [25] RT-PCR: [25]	–

## Discussion

With a poor prognosis, gastric cancer poses a great burden particular in eastern Asia countries. [27]. However, hitherto there is no clinically approved biomarker so far for an early intervention [28]. CD133, as a potential prognostic marker has been identified in a number of cancers [29]. In the present study, CD133 in GC patients appeared to have a median expression rate of 44.8% (15.2%–57.4%) by IHC solution based on Asian patients. With reference to clinicopathological features, CD133 expression was closely associated with tumor size, N stage, lymphatic/vascular infiltration, as well as TNM stage (by IHC method). It is believed that the correlation of lymph node metastasis and TNM stage were of high prognostic value [30]–[31]. In accordance with some other types of malignances, CD133 overexpressed GC patients was of lower 5-year overall survival in comparing to negative ones.

CD133, also termed prominin-I in a family of 5-transmembrane glycoprotein, was the first to be identified in mice by Weigmann in 1997 [32]. It was originally classified as a marker of primitive haematopoietic and neural stem cells. Also, CD133 are widely used as a candidate of cancer stem cell marker in gastrointestinal tumors. It is intriguing that cultured in serum-free medium supplemented with EGF and bFGF, one CD133-positve colon cancer cell is able to form a cancer sphere however CD133-negative ones are not. While injected to severe combined immune deficiency (SCID) mouse, only CD133-positive colon cancer cells could form tumors [33]. This exclusive self-renew and tumorgenecity capacity strongly suggested it to be a potential colon cancer stem cell marker. Caner stem cell is believed to play a key role in initiating, progression, metastasis and recurrence of cancer, and eventually influence the overall survival of cancer patients. Study by Horst et al. found that overexpression of CD133 was detected in 28.4% of stage II-A colon cancer [34]. Combined analysis of CD133 and β-catenin identified an elevated risk in stage II-A colon cancer patients. For those cases characterized by lack of nuclear β-catenin regulation and high CD133 expression, a vastly declined cancer-specific and disease-free survivals were found compared to their counter partners. Although the relationship between CD133 and gastric cancer stem cell remains obscure, emerging evidence have disclosed the potential correlation [35].

In line with Tratuzumab targeted therapy, the ToGA trial indicated a Combination of tranterzumab with chemotherapy significantly improved the overall survival of HER2-positive gastric cancer patients, compared with chemotherapy alone [36]. The treatment is based on the rationale that approximately 20% GC patients with HER2 overexpression [37]. Cancer stem cell could be a potential target for therapeutics [38]. Given the insights from the strong correlations between CD133 and prognosis/clinicopathological features, it could help in the development of strategies to gastric cancer. And *in vitro* test have shown that, cytotoxic drug is able to induce cell apoptosis by specifically combined to surface antigen CD133, and therefore inhibit the growth of gastric cell line [39]. Nevertheless the clinically translational potentials warrant further investigation.

Though the results from our study pointed to a positive correlation between CSC marker CD133 and overall survival, there are still some controversies. First of all, CD133 is still a candidate but not a definite CSC marker, for example, results from Rocco A [40] showed that neither CD133 nor CD44 can be an eligible marker to isolate cancer stem cells; some other studies revealed that not only CD133-positive, but also CD133-negative ones can initiate a tumor [41]–[42]. And many scientists insist on combined markers for the identification of CSC now, i.e., CD44+CD24- for breast CSC, CD44+CD54- for gastric CSC [43]–[44]. Secondly, for gastric cancer and some other cancer patients whose tumor tissue over express a CSC marker, their recurrence rate or overall survival was not always worse than the negative ones [45]–[47]. Thus more prospective studies are needed to draw a definite conclusion.

This systematic review has some limitations. First, the number of included studies, as well as the included GC patients in each study, is relatively small. Secondly, all those seven studies are based on Asian population, including 1 from Japan, 1 from Singapore and the rest 5 from China. It is believed that distinct site difference exists in gastric tumor between western and eastern populations. In Asia, the majority gastric cancer present in the distal stomach whereas cardia cancer is the predominately cancer type in western countries. The etiology, biology features, and prognosis are of significant difference between the two categories. As a result, whether the overexpression rate of CD133 as well as its function in Western patients is identical with Asian ones is still unknown, because there isn’t any study on this topic based on Western populations until known. Higher quality studies with more volume of patients are needed to clarify the clinicopathological factors associated with CD133-positive gastric cancer and its impact on survival outcomes. More importantly, an improved knowledge in cancer biology and cancer stem cell can further potentiate the induction of new targeted therapeutics.
