# Correction: Prognostic Value of Cancer Stem Cell Marker CD133 Expression in Gastric Cancer: A Systematic Review

**DOI:** 10.1371/annotation/80c50c32-16b8-4099-98e4-af72154192b3

**Published:** 2014-01-16

**Authors:** Lei Wen, Xin-Zu Chen, Kun Yang, Zhi-Xin Chen, Bo Zhang, Jia-Ping Chen, Zong-Guang Zhou, Xian-Ming Mo, Jian-Kun Hu

In the list of references, 29 is incorrect. Reference 29 should read, "Coco C, Zannoni GF, Caredda E, Sioletic S, Boninsegna A, et al. (2012) Increased expression of CD133 and reduced dystroglycan expression are strong predictors of poor outcome in colon cancer patients. J Exp Clin Cancer Res. 31: 71." 

Additionally, the affiliation for Dr. Xian-Ming Mo should be Laboratory of Stem Cell Biology, State Key Laboratory of Biotherapy, West China Hospital, Sichuan University, Chengdu, Sichuan Province, China.

